# Institutional Needs

**Published:** 1921-09-03

**Authors:** 


### Institutional Needs.

### "Harpic": A New Lavatory Cleanser.

THIS is a cleansing disinfectant powder which is claimed to be the only safe lavatory cleanser. It eliminates any necessity for the use of spirits of salt, and the manufacturers guarantee that it will remove all stains, no matter how bad, and by regular application keep lavatories thoroughly disinfected and spotless. Certainly the trial we have given it shows it to be thoroughly efficacious in its results. It has the further recommendation that it is easy to use, whilst the cost is said to work out at less than one penny a month for each lavatory pan. It is non-inflammable and contains no scheduled poison. In addition to lavatory pans, it will also clean sinks, baths, and other vitrified ware. The only adverse comment we have heard of it comes from a practical housekeeper, who, though thoroughly satisfied with it as a cleanser, takes objection to the name, which, she contends, is neither euphonious nor easy to remember. That is a point the manufacturers (the Harpic Manufacturing Co., 46-48 Goswell Road, E.C.) may like to ponder.

